# Molecular Insights into the Genetic Diversity of *Hemarthria compressa* Germplasm Collections Native to Southwest China

**DOI:** 10.3390/molecules191221541

**Published:** 2014-12-22

**Authors:** Zhi-Hui Guo, Kai-Xin Fu, Xin-Quan Zhang, Shi-Qie Bai, Yan Fan, Yan Peng, Lin-Kai Huang, Yan-Hong Yan, Wei Liu, Xiao Ma

**Affiliations:** 1 Department of Grassland Science, Animal Science and Technology College, Sichuan Agricultural University, Ya’an 625014, China; E-Mails: guozhihui-2008@163.com (Z.-H.G.); linzhuyan@126.com (K.-X.F.); zhangxq@sicau.edu.cn (X.-Q.Z.); baishiqie@yeah.net (S.-Q.B.); pengyanlee@163.com (Y.P.); huanglinkai@sicau.edu.cn (L.-K.H.); yanyanhong3588284@126.com (Y.-H.Y.); lwgrass@126.com (W.L.); 2 Sichuan Academy of Grassland Science, Chengdu 611731, China; 3 Chongqing Municipal Institute of Animal Husbandry, Chongqing 400039, China; E-Mail: cq_fy001@163.com

**Keywords:** *Hemarthria compressa* L., start codon targeted polymorphism (SCoT), genetic diversity, geographic groups, genetic differentiation

## Abstract

Start codon targeted polymorphism (SCoT) analysis was employed to distinguish 37 whipgrass (*Hemarthria compressa* L.) clones and assess the genetic diversity and population structure among these genotypes. The informativeness of markers was also estimated using various parameters. Using 25 highly reproducible primer sets, 368 discernible fragments were generated. Of these, 282 (77.21%) were polymorphic. The number of alleles per locus ranged from five to 21, and the genetic variation indices varied. The polymorphism information content (PIC) was 0.358, the Shannon diversity index (H) was 0.534, the marker index (MI) was 4.040, the resolving power (RP) was 6.108, and the genotype index (GI) was 0.782. Genetic similarity coefficients (GS) between the accessions ranged from 0.563 to 0.872, with a mean of 0.685. Their patterns observed in a dendrogram constructed using the unweighted pair group method with arithmetic mean analysis (UPGMA) based on GS largely confirmed the results of principal coordinate analysis (PCoA). PCoA was further confirmed by Bayesian model-based STRUCTURE analysis, which revealed no direct association between genetic relationship and geographical origins as validated by Mantel’s test (*r* = 0.2268, *p* = 0.9999). In addition, high-level genetic variation within geographical groups was significantly greater than that between groups, as determined by Shannon diversity analysis, analysis of molecular variance (AMOVA) and Bayesian analysis. Overall, SCoT analysis is a simple, effective and reliable technique for characterizing and maintaining germplasm collections of whipgrass and related species.

## 1. Introduction

Whipgrass (*Hemarthria compressa* L.) is a warm-season perennial creeping grass of the tribe Andropogoneae in the family Poaceae. Whipgrass is one of the most important and widely utilized forages plants in southern China due to its high forage yield, regeneration, fast growth and adaptability to hot, humid conditions [1]. The large morphological variations within wild whipgrass collections produce great potential for breeding selection [2]. Over the last 50 years, studies by Sichuan Agricultural University have resulted in the release of three cultivars: “Guangyi”, “Chonggao” and “Yaan”. These cultivars have been widely used in the Yangtze River and play an essential role in animal husbandry and environmental sustenance [1,3].

Simple, accurate and rapid genotype identification is extremely important for germplasm characterization and the practical breeding of vegetatively propagated forage species such as whipgrass and many other warm-season perennial grasses. Molecular markers can be reliably used for cultivar identification, biodiversity analyses, phylogenetic studies and other applications, whereas plant morphological traits are limited in number and vulnerable to environmental impacts [4]. The marker system that is selected for use in a particular application depends on its ease of use and the particular objectives of the investigation. It has been suggested that the measurement of genetic diversity by molecular markers for breeding purposes should be based on functionally characterized genes or targeted genes because these may reflect functional polymorphisms [5]. A variety of marker systems, including Inter-Single Sequence Repeats (ISSR) [6], Sequence Related Amplified Polymorphism (SRAP) [7] and Expressed Sequence Tag-Simple Sequence Repeat (EST-SSR) [8] markers revealed significant levels of genetic variation and the relationship between whipgrass accessions from Southwest China. Taken together, these studies allowed the identification of genetically distinct subgroups depending on marker types, and inferred that clustering patterns has a weak correlation with geographical origins or ecotypes of wild germplasm. Recently, Collard and Mackill [9] proposed a new dominant molecular marker in rice, which is based on SPAR (single primer amplification reaction), and it was called the start codon targeted (SCoT) polymorphism. Because the ATG translation start site and its flanking sequences are conserved in plant genes, a single primer was designed and used to amplify the genomic region. As a new molecular marker method based on PCR technology, SCoT has many advantages. The technique is simple, low cost and highly polymorphic and provides extensive genetic information; moreover, its primers are universal in plants, as validated in genetic diversity studies of peanut [10], mango [11], grape [12], chickpea [13],* Cleome gynandra* [14] and wheat [15]. 

The purpose of this study was to determine the potential use of SCoT markers in whipgrass clones and to evaluate the level of genetic variation among 37 wild clones collected from four geographic regions in southwest China and three whipgrass cultivars. Although molecular marker analyses of whipgrass are available [6,7,8,16], this is the first molecular marker analysis of whipgrass to utilize SCoT markers. An extensive analysis based on various methods was implemented in the present study.

## 2. Results and Discussion

### 2.1. Polymorphisms Detected Using SCoT Markers

A total of 368 bands were generated using 25 prescreened SCoT primer sets with 37 whipgrass genotypes. Of these bands, 282 were polymorphic (76.63%). The number of bands varied from 5 (SCoT87) to 24 (SCoT21 and SCoT84) per primer, corresponding to an average of 8.4 amplicons and 11.28 polymorphisms per primer, respectively. The percentage of polymorphic bands (PPB) ranged from 52.94% for primer SCoT60 to 100% for the following primers: SCoT5, SCoT7, and SCoT87 (Table 1). None of the amplification profiles generated by any of the primer was found to be identical to all the accessions. A number of marker attributes such as Shannon’s diversity index (H), polymorphism information content (PIC), marker index (MI), and resolving power (Rp) together with genotype index (GI) were used to evaluate the informativeness of the primer sets. The SCoT primer number 21, 43, 55 and 84 generated the most informative band profile as it identified all of 37 accessions analyzed. Subsequent informative primer was 90 and 93 that discriminated 36 out of 37 accessions. Primer 87 was least informative as it distinguished only 14 accessions. Shannon index (H) ranged from 0.0423 to 0.617, with a mean of 0.534; the PIC value ranged from 0.267 to 0.429 with an equilibration of 0.358 across all genotypes. The average values of MI, Rp and GI were 4.040, 6.108 and 0.782, respectively.

**Table 1:** Sequences of the SCoT primers and statistics for the amplification of the whipgrass clones.

Primer Code	Sequence (5'→3')	TNB	NPB	PPB (%)	H	PIC	MI	Rp	GI
SCoT4	caacaatggctaccacct	12	9	75.00	0.524	0.347	3.121	4.703	0.784
SCoT5	caacaatggctaccacga	6	6	100.00	0.518	0.340	2.039	2.811	0.459
SCoT7	caacaatggctaccacgg	10	10	100.00	0.564	0.385	3.845	5.892	0.838
SCoT8	caacaatggctaccacgt	9	7	77.78	0.459	0.294	2.060	2.919	0.486
SCoT10	caacaatggctaccagcc	14	11	78.57	0.520	0.343	3.778	5.243	0.811
SCoT21	acgacatggcgacccaca	24	19	79.17	0.564	0.385	7.322	11.405	1.000
SCoT23	caccatggctaccaccag	17	11	64.71	0.558	0.376	4.131	6.000	0.892
SCoT25	accatggctaccaccggg	20	18	90.00	0.514	0.340	6.124	8.919	1.000
SCoT31	ccatggctaccaccgcct	10	8	80.00	0.477	0.310	2.478	3.351	0.541
SCoT32	ccatggctaccaccgcac	12	8	66.67	0.570	0.390	3.118	5.135	0.757
SCoT33	ccatggctaccaccgcag	13	9	69.23	0.551	0.374	3.369	5.189	0.757
SCoT43	caatggctaccaccgcag	20	17	85.00	0.517	0.346	5.876	9.081	1.000
SCoT46	acaatggctaccactgag	11	7	63.64	0.617	0.429	3.001	4.865	0.649
SCoT47	acaatggctaccactgcc	15	8	53.33	0.593	0.408	3.261	5.297	0.730
SCoT48	acaatggctaccactggc	11	8	72.73	0.520	0.346	2.770	4.270	0.622
SCoT52	acaatggctaccactgca	20	16	80.00	0.519	0.346	5.537	8.649	0.946
SCoT55	acaatggctaccactacc	15	12	80.00	0.532	0.356	4.275	6.216	1.000
SCoT57	acaatggctaccactacg	10	7	70.00	0.543	0.368	2.577	4.108	0.568
SCoT59	acaatggctaccaccatc	14	10	71.43	0.519	0.349	3.495	5.622	0.838
SCoT60	acaatggctaccaccaca	17	9	52.94	0.570	0.386	3.471	5.297	0.757
SCoT83	acgacatggcgaccagcg	17	12	70.59	0.424	0.267	3.205	4.378	0.784
SCoT84	acgacatggcgaccacgt	24	21	87.50	0.546	0.370	7.778	12.324	1.000
SCoT87	accatggctaccaccggt	5	5	100.00	0.536	0.357	1.785	2.595	0.378
SCoT90	ccatggctaccaccggca	21	15	71.43	0.561	0.379	5.683	8.378	0.973
SCoT93	accatggctaccagcgca	21	19	90.48	0.541	0.363	6.890	10.054	0.973
Min.	-	5	5	52.94	0.424	0.267	1.785	2.595	0.378
Max.	-	24	21	100.00	0.617	0.429	7.778	12.324	1.000
Means	-	14.72	11.28	77.21	0.534	0.358	4.040	6.108	0.782
Total	-	368	282	-	-	-	100.996	152.703	1.000

TNB: total number of bands; NPB: number of polymorphic bands; PPB: percentage of polymorphic bands; H: Shannon diversity index; PIC: polymorphism information content; MI: marker index; GI: genotype index; Rp: resolving power.

A positive linear relationship (*p* < 0.01) was observed between GI and Rp (*r* = 0.860), between Rp and MI (*r* = 0.993) and between GI and MI based on average diversity (*r* = 0.870). However, these three parameters were found no clear correlation with PIC. Nevertheless, a positive linear relationship (*p* < 0.01) linear relationship was observed between PIC and H (*r* = 0.998).

### 2.2. Genetic Similarity and Cluster Analysis

**Figure 1:**
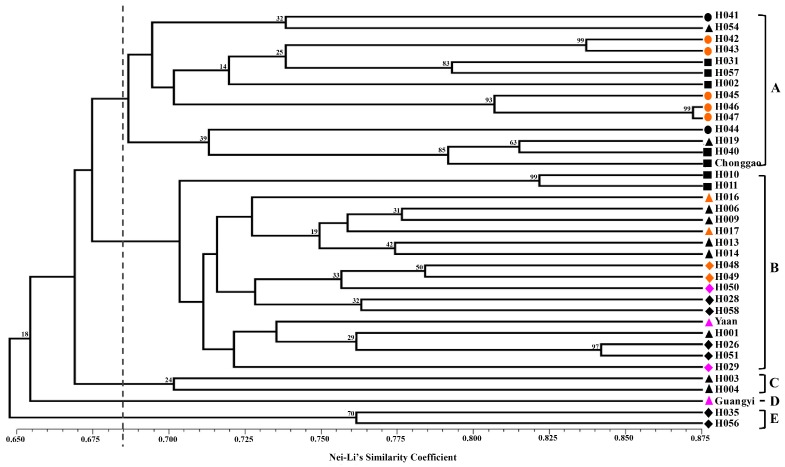
Dendrogram of genetic relationships between whipgrass clones determined by SCoT. The numbers on the branches were verified by bootstrapping analysis to assess the robustness of the dendrogram topology from 1000 replicates. The symbols represent geographic groups in the cluster tree as ● Clones from Guizhou (GZ), ♦ Clones from Yunnan and Liangshan (YL), ▲ Clones from Chengdu Plain (CDL), and ■ Clones from Chongqing (CQ). The three different colors (orange, black and pink) represent morphological types (I, II and III) in the cluster tree separately.

Nei-Li similarity coefficients (GS) ranged from 0.563 (H019* vs.* H056) to 0.872 (H046* vs.* H047). These results indicate relatively high genetic variability among the examined whipgrass clones. In the unrooted dendrogram made on the basis of similarity index, all 37 accessions grouped into five clusters (A, B, C, D and E) with a mean similarity threshold of 0.685, whereas the corresponding bootstrapping values of branches were relatively low (Figure 1). Cluster A included 14 accessions from Chongqing (CQ) and Guizhou (GZ). Cluster B comprised 18 accessions from Chengdu Plain (CDL), Yunnan and Liangshan (YL). The remaining five accessions belonged to clusters C, D and E. No clear distinctions between the geographical localities of the samples could be drawn. In Figure 1, the distribution of morphological types (I, II and III), which was showed by three colors (orange, black and pink), was not associated with the division of the cluster by UPGMA. 

### 2.3. Principal Coordinate Analysis (PCoA)

**Figure 2:**
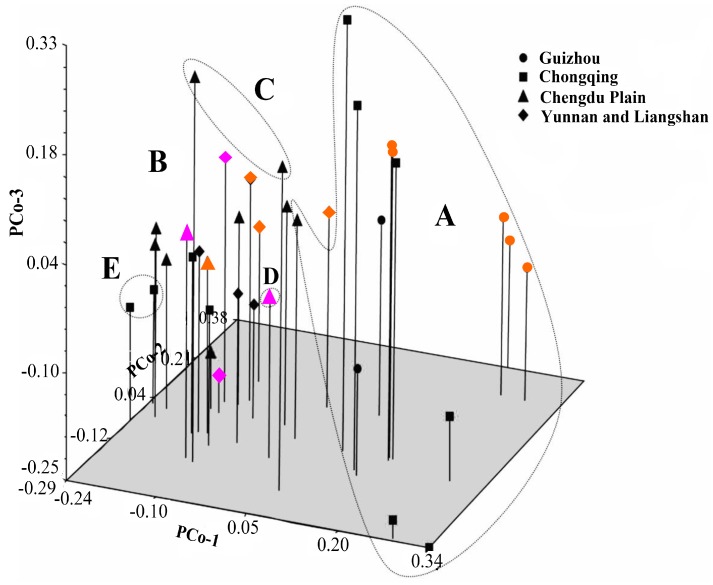
Dimensions plot of the principal coordinate analysis of whipgrass clones. Genetic relationships are depicted among the 37 whipgrass genotypes by the first three components (PCo-1, PCo-2, and PCo-3) derived from PCoA of the SCoT data. The geographic origin of each clone is indicated by the symbols listed at the right; ▲: Chengdu Plain (CDL); ●: Guizhou (GZ); ♦: Clones from Yunnan and Liangshan (YL); and ■: Chongqing (CQ). The three different colors (orange, black and pink) represent morphological types (I, II and III) in the cluster tree separately.

To better understand the relationships among the accessions, PCoA was conducted using the genetic similarities data set. PCoA was largely congruent with the assignments generated by UPGMA clustering (Figure 2). The first three principal axes accounted for 9.46%, 5.81%, and 5.55% of the total variation, respectively. These results indicate that the multidimensional nature of SCoT variation is low and that most of the variation observed was due to genotypes per geographic group. The accessions belonging to the A group (as inferred by UPGMA clustering) were mainly distributed in the right portion of the resulting plot. The remaining four groups (B, C, D, E) were distributed in the left portion of the plot. Both clustering and PCoA failed to group accessions with similar geographic origins and/or morphological types together.

### 2.4. Genetic Structure Analysis

**Figure 3:**
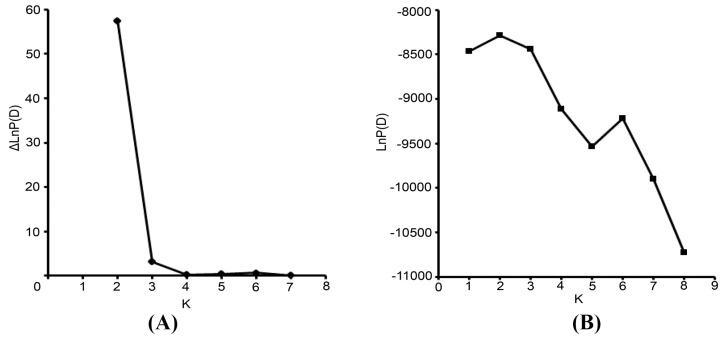
Two different methods for determining the optimal value of K. (**A**) The Second-order statistics (ΔK) method developed by Evanno* et al.* [17]. (**B**) The *ad hoc* procedure described by Pritchard* et al.* [18].

**Figure 4:**
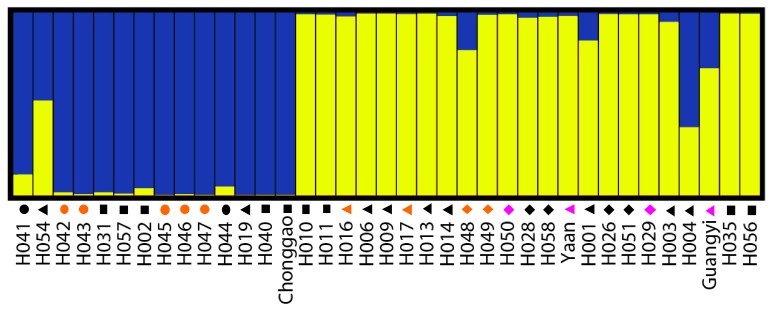
Estimated population structure for whipgrass clones (K = 2). Each individual is represented by a single color line. The greater proportion of a color (blue or gold), the greater the possibility that the represented individual belongs to the group indicated by that color. Cluster a is indicated by the color gold and Cluster b is indicated by the color blue. The symbols represent geographic groups in the cluster tree as ● Clones from Guizhou (GZ), ♦ Clones from Yunnan and Liangshan (YL), ▲ Clones from Chengdu Plain (CDL), and ■ Clones from Chongqing (CQ). The three different colors (orange, black and pink) represent morphological types (I, II and III) in the cluster tree separately.

The pattern of genetic diversity and population structure was further analyzed for the complete set of 37 accessions with a Bayesian-based approach implemented using the STRUCTURE program. Estimated likelihood values for a given K in ten independent runs yielded consistent results; however, the distribution of LnP(D) did not show a clear mode for the true K. This result is expected when factors such as departures from Hardy–Weinberg equilibrium are present. Therefore, an ad hoc quantity (ΔLnP(D)) was used to overcome the difficulty in interpreting real K values [17]. Using this approach, an identifiable peak indicated the true value of K based on ΔLnP(D). Fortunately, the highest values (K = 2) of LnP(D) and ΔLnP(D) for the 37 accessions were identical in this study. We therefore chose a value of K = 2 confirm for the final analysis (Figure 3 and Figure 4). In other words, accessions were separated into two clusters or two types of genetic backgrounds (Figure 4). Each individual was represented by a single color line, and each cluster was represented by a color. The greater proportion of a color that an individual received, the greater the possibility that the individual belonged to the corresponding cluster. As shown in Table 2, Cluster a (indicated by the color gold in Figure 4) included 22 accessions; most of these accessions were from CDP and YL. Cluster b (indicated by the color blue in Figure 4) included 14 accessions, mostly from GZ and CQ.

Using the same initial conditions, independent repeats at the same inferred value K might yield a different individual membership coefficients matrix (Q-matrix). Of 37 accessions, 36 could be assigned to clusters based on a 60% membership threshold. Thus, more than 97.3% of the accessions were considered to have a relatively simple parentage (Table 2). 

**Table 2:** Distribution of the Q value of germplasm of different origins in the 2 inferred clusters.

Geographic Groups	No.	Cluster a (Q_1_ > 0.6)	Cluster b (Q_2_ > 0.6)	Q ≤ 0.6
GZ	7	0	7	0
CQ	9	4	5	0
CDP	13	10	2	1
YL	8	8	0	0
Total	37	22	14	1

GZ: Guizhou; CQ: Chongqing; CDP: Chengdu Plain; YL: Yunnan and Liangshan.

### 2.5. Genetic Divergence in Geographic Groups

#### 2.5.1. Shannon Diversity Analysis

The Shannon diversity index of the four geographic groups ranged from 0.3964 to 0.5042, with an average of 0.4516. The genetic diversity (84.57%) was much higher within groups than between groups (15.43%). The results from the variance analysis indicated that there were significant differences in intra-group diversity in CQ and CDP; however, the intra-group diversity in CQ and CDP was significantly greater than the intra-group diversity in the GZ and YL groups (Table 3). 

**Table 3:** Partitioning of the genetic variation into within- and between- geographic groups analyzed by the Shannon diversity index.

Geographic Groups	H_zone_ (S.D.)	H_A_ (S.D.)	H_W_ (S.D.)	H_A_/H_W_	(H_W_ − H_A_)/H_W_
GZ	0.3964 (0.2736) ^B^	0.4516 (0.0545)	0.5340 (0.1529)	0.8457	0.1543
CQ	0.4921 (0.2248) ^A^				
CDP	0.5042 (0.2028) ^A^				
YL	0.4136 (0.2620) ^B^				

GZ: Guizhou; CQ: Chongqing; CDP: Chengdu Plain; YL: Yunnan and Liangshan. H_zone_: the genetic variation within groups; H_A_: the average genetic variation within groups; H_W_: the total genetic variation; H_A_/H_W_: the proportion of genetic variation within groups; (H_W_ − H_A_)/H_W_: the proportion of genetic variation between groups (Uppercase letters ^A^ and ^B^ indicate significant differences at the 1% level).

#### 2.5.2. Analysis of Molecular Variance (AMOVA)

AMOVA of the four geographic groups revealed that most of the variation occurred the intra-group (93.3% of the total variation), and only 6.66% could be attributed to differences between-groups (Table 4). Pairwise estimates of Φ_PT_ indicated a different degree of variation between the geographic groups, with values ranging from 0.048 (between CDP and YL) to 0.153 (between GZ and YL) (Table 5). 

**Table 4:** Analysis of molecular variance (AMOVA) of the geographic groups.

Source of Variation	df	SS	Ms	Est. Var.	PVC (%)	Φ_PT_	*p* Value
Between groups	3	243.261	81.087	3.514	6.66%	0.067	<0.0001
Within groups	33	1625.010	49.243	49.243	93.34%		
Total	36	1868.270		52.756	100%		

df: degree of freedom; SS: square deviation; Ms: mean square deviation; Est. Var.: estimated variance; PVC(%): percentage of variance component; Φ_PT_: coefficient of genetic differentiation.

**Table 5:** Pairwise Φ_PT_ values among geographic groups.

Geographic Groups	GZ	CQ	CDP	YL
GZ	0.000			
CQ	0.070	0.000		
CDP	0.107	0.014	0.000	?
YL	0.153	0.054	0.048	0.000

GZ: Guizhou; CQ: Chongqing; CDP: Chengdu Plain; YL: Yunnan and Liangshan.

The overall Φ_PT_ value was 0.067, confirming that geographic distribution had little effect on the diversity of whipgrass genotypes. 

#### 2.5.3. Bayesian Inference

The Bayesian approach provided the posterior mean, standard deviation, and 95% confidence interval for each of the four available models. The smallest mean DIC was obtained under the *full* model, suggesting that it was the most suitable model for these data. For the four geographic groups, this model (DIC 3967.02) generated a mean *θ^B^* of 0.0613 ± 0.0098 (95% confidence intervals of 0.0423 and 0.0808) and a G_ST_-B of 0.0464 ± 0.0073 (95% confidence intervals of 0.0325 and 0.0607). The Bayesian estimates of average panmictic heterozygosity (H_S_) are shown in Table 6. At the population level, the total data indicated that the lowest and highest levels of genetic variation occurred in the YL group (H_S_ = 0.3614) and CQ group (H_S_ = 0.3696), respectively. At the species level, genetic variation was 0.3841 for the total data. In total, these results demonstrate that the *full* model yielded estimates of F_ST_ in concordance with those previously described in the extension of the AMOVA (Table 4), supporting the conclusion that most variability occurred within rather than between geographical groups. Moreover, the DIC value (DIC = 3967) under the *full* model was 51 units lower than under the ƒ = 0 model (DIC = 4018). This result indicates deviation from Hardy-Weinberg equilibrium. The full model was also favored over the *θ* = 0 model (DIC = 4261), indicating significant population structure [19].

**Table 6:** Partitioning of the genetic variation into within- and between-geographic groups analyzed by Bayesian inference (*full* model).

Geographic Groups	H_S_ (S.D.)	H_S_ (S.D.)	H_T_ (S.D.)	H_S_/H_T_	G_ST_-B = (H_T_ − H_S_)/H_T_
GZ	0.3621 (0.0109)	0.3663 (0.0094)	0.3841 (0.0082)	0.9536	0.0464
CQ	0.3696 (0.0087)				
CDP	0.3720 (0.0090)				
YL	0.3614 (0.0119)				

GZ: Guizhou; CQ: Chongqing; CDP: Chengdu Plain; YL: Yunnan and Liangshan. H_S_: the genetic variation within groups; H_S_: the average genetic variation within groups; H_T_: the total genetic variation; H_S_/H_T_: the proportion of genetic variation within groups; (H_T_ − H_S_)/H_T_: the proportion of genetic variation between groups. G_ST_-B: coefficient of genetic differentiation.

### 2.6. Discussion

#### 2.6.1. Genetic Diversity in the Whipgrass Collection

Genetic diversity analysis is vital for the management of entire collections and breeding programs. The efficiency of a marker technique in discriminating genotype depends largely upon the polymorphism it can detect. The SCoT technique has been used successfully for the DNA fingerprinting, characterization of genetic variation and phylogenetic studies in some cultivated plants [9,10,11,12,13,14,15]. The results obtained using 25 screened primers demonstrated that SCoT markers can be effectively used to estimate the genetic diversity of and to judge the genotype of wild whipgrass collections with multiple morphological types, as shown by the various genetic diversity indices (PIC = 0.358, H = 0.534, MI = 4.040, RP = 6.108 and GI = 0.782). The percentage of polymorphic bands (PPB), a major genetic diversity index, also indicated rich diversity in whipgrass genotypes (77.21%), in contrast to the results of ISSR (84.2%) [6], SRAP (91.5%) [7] and EST-SSR (80.4%) [8] analysis. The discrepancy in the rate of diversity between marker techniques may be due to differences in the source of the detected diversity. Each technique targets different regions of the genome [20]. In principle, SCoT is similar to RAPD and ISSR because a single primer is used as the forward and reverse primers [9]. PIC can be determined based on both the number and frequency of amplified fragments to measure the discriminatory power of a genetic marker system [21]. Although the average PIC (0.385) in this study is lower than that determined using SCoT for the vegetative plant potato (0.40) [20] and that determined by EST-SSR for whipgrass (0.47) [8], the PIC still confirmed the good discriminatory capacity of the primers as a maximum PIC values of 0.5 for dominant markers[22]. In addition, primer Rp and MI based on band informativeness and diversity index, are also the parameters used for identifying primers with high discrimination ability. Significant correlations between Rp, MI and GI, which indicate the ability of primers to distinguish between genotypes, revealed strong positive linear relationships. These linear correlations indicated that it is possible to estimate the number of genotypes simply by calculating Rp or MI rather than PIC of a primer. In present work, the average genotype index (GI) of 0.782 across 25 primers means 29 out of 37 accessions could be identified by per primer. Besides, SCoT showed more informative and effective than other dominant markers such as RAPD and ISSR for diagnostic fingerprinting genotypes or varieties in chickpea [13], *Morinda tomentosa* [23], and tetraploid potato [20]. This suggests that SCoT technique is highly efficient and valuable in genotyping of whipgrass germplasm.

#### 2.6.2. Genetic Relationships in the Whipgrass Collection

The two different methods of multivariate analysis (PCoA and UPGMA analysis) applied to group individuals in this study sort data on different calculation bases; therefore, they can be used to elucidate relationships comparatively. The UPGMA-based phenogram and principal ordinate analysis displayed similar patterns. UPGMA analysis assumes a constant evolutionary rate among accessions. This assumption can be violated when comparing samples collected in the wild with those selected by humans. However, the purpose of the present study was to investigate the level of genetic variation rather than to determine which accession evolved from the others. Moreover, the STRUCTURE analysis grouped whipgrass accessions into two major clusters based on the Bayesian Method (Figure 3 and Figure 4). The degree of admixture (α) was determined from the SCoT data. When α is near zero, most individuals are essentially from one population. Conversely, when α is greater than 1, most individuals are admixed [17,24]. The relatively small value of α (α = 0.082) suggests that most accessions originated from one primary ancestor [24].

Ecological and geographical conditions are important factors affecting plant growth, development and distribution. Most accessions with geographical proximity were grouped into different clusters, indicating that there was no direct association between genetic divergence and geographic origins, as validated by Mantel’s test (*r* = 0.2268, *p* = 0.9999). These results correspond with studies of whipgrass collection from southwest China by using ISSR [6], SRAP [7] and EST-SSR [8], in which no clear relationship could be detected between groups and geographical origin or morphotype of the accessions because intermixing of accessions with different eco-geographical sites or morphological features were across most clusters. The reliability of association between genetic divergence and geographic origins in previous studies [6,7,8] was still doubtful, due to the absence of precise quantitative verifications such as Mantel’s test or bootstrap analysis. In summary, genetic group division of whipgrass accessions in present work by SCoT cannot be defined clearly on the basis of geographical origin or morphotype, which was equivalent to previous studies by other dominant markers. This result also were found to be in broad agreement with STRUCTURE analysis, AMOVA, Bayesian inference and Shannon diversity analysis, which demonstrated that the variation within groups was much greater than that between groups. The reasons why genetic groups from cluster analysis had no association with geographic origin of whipgrass accessions can be attributed to a number of causes. First, whipgrass clones are reproduced in different proportions due to its strong asexual reproduction capacity and ecological adaptability to natural factors (e.g., river flow) and human activities (such as introduction, domestication and germplasm exchange). Second, natural hybridization between whipgrass clones occurs, although the seed setting rate is low. Such hybridizations produce new genotypes via genetic recombination, which might increase the distribution of genetic diversity and the geographic origin of complexity [25]. Third, gene mutations might occur in whipgrass as part of adaptations to the environment under selective pressures resulting from the natural environment or human activities [26].

The dendrogram cannot distinguish the accessions into morphological types. Several factors might explain this lack of correlation between morphological traits and molecular markers. First, the number of selected primers did not cover the vast area of the whipgrass genome. Second, morphological variation is strongly associated with characteristic regional environmental conditions. Finally, the morphological similarities observed might be due to different combinations of alleles producing similar phenotypes, which might result in morphological similarities or differences that are not proportional to underlying genetic differences [27].

#### 2.6.3. Genetic Differentiation in Geographic Groups

Genetic differentiation can be affected by many factors, including mutation, genetic drift, the breeding system, the mating system, gene flow and selection [28,29], geographical distribution and genetic marker types. Genetic differentiation in the four geographic groups tested by AMOVA, Bayesian inference and Shannon diversity analysis demonstrated that the variation within groups was much greater than that between groups. In other words, these results implied little differentiation between groups, coinciding with SRAP [7] and EST-SSR [8].

Bayesian inference demonstrated that all geographic groups deviated from the genetic equilibrium state. Therefore, software based on Hardy-Weinberg equilibrium assumptions, such as POPGENE [30], should not be used to calculate genetic heterozygosity, the population differentiation coefficient and other parameters. The genetic differentiation coefficients of the geographic groups determined by the AMOVA, Bayesian and Shannon analyses were Φ_PT_ = 0.067, G_ST_-B = 0.0464, (H_W_ − H_A_)/H_W_ = 0.1543, respectively. The results from these three methods exhibited discrepancies due to the differing principles of the methods. Shannon analysis is based on amplified phenotypic frequency bands [31], and AMOVA is based on the variance components and the significance level of dominant marker data for the degree of haplotype divergence [32]. Bayesian inference cannot predict the degree of population inbreeding or estimate the proportion of genotypes within populations under Hardy-Weinberg equilibrium. The dominant marker information is used to calculate population genetic diversity (H_S_) and population genetic differentiation (G_ST_-B) [19]. The three types of analytical methods suggested that the geographic genetic diversity within groups was much greater than that between groups. In addition, high levels of genetic variation were indirectly deduced among the tested genotypes. Although the actual contribution of human activity to the rate of gene flow is unknown, the low levels of differentiation among geographical groups might reflect human activities in different regions resulting in germplasm exchange.

## 3. Experimental Section

### 3.1. Plant Material

**Figure 5:**
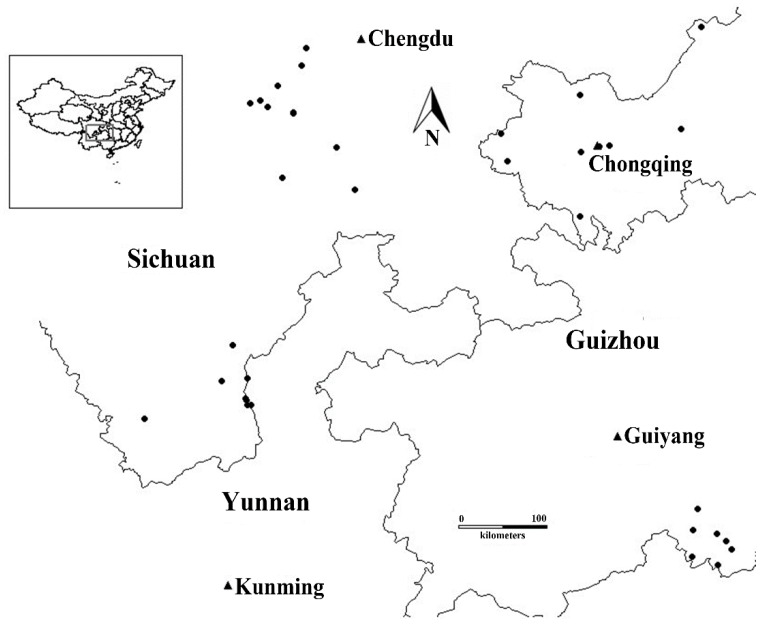
Map showing the geographical locations of the 37 whipgrass clones in China, with spots indicating the collection sites.

A total of 37 whipgrass (*H. compressa* L.) germplasms, including three registered varieties, were analyzed in the present study (Table 7 and Figure 5); these accessions were collected from southwest China. Based on geographic origin and ecological environment, the materials were divided into the following four geographic groups: Chengdu Plain (CDL), Chongqing (CQ), Guizhou (GZ), and Yunnan and Liangshan (YL) [33]. Based on a previous study of the morphological traits of whipgrass collections from southwest China [2], the germplasms were classified into the following three categories: (I) high-erect type (thin and long leaves, long internodes and erect plants); (II) fine-low type (thin and short leaves, fine and stoloniferous stems, short internodes and low-creeping plants); and (III) thickset-low type (wide and long leaves, short internodes, stocky-stoloniferous stems and creeping plants) (Table 7). Using the root tip squash method [34], we found that most of the whipgrass accessions used in the present study were hexaploid (2*n* = 6x = 54); only four accessions were tetraploid (2*n* = 4x = 36). 

**Table 7:** Source of the clones of *H. compressa*.

No.	Code	Origin	Latitude (N)	Longitude (E)	Altitude (m)	Geographic Groups	Morphological Types †	Ploidy+
1	H041	Dushan, Guizhou	25°20'18"	107°28'41"	930	GZ	II	6x
2	H042	Dushan, Guizhou	25°36'20"	107°29'38"	950	GZ	I	6x
3	H043	Dushan, Guizhou	25°49'23"	107°32'31"	970	GZ	I	6x
4	H044	Dushan, Guizhou	25°34'29"	107°44'13"	820	GZ	II	6x
5	H045	Libo, Guizhou	25°29'58"	107°49'45"	890	GZ	I	6x
6	H046	Libo, Guizhou	25°24'33"	107°53'25"	420	GZ	I	6x
7	H047	Libo, Guizhou	25°15'08"	107°44'35"	410	GZ	I	6x
8	H002	Rongchang, Chongqing	29°24'06"	105°34'59"	600	CQ	II	6x
9	H010	Nanshan, Chongqing	29°33'24"	106°37'56"	420	CQ	II	6x
10	H011	Hechuan, Chongqing	30°04'36"	106°19'32"	270	CQ	II	6x
11	H031	Yuzhong, Chongqing	29°33'13"	106°31'30"	180	CQ	II	6x
12	H035	Liangping, Chongqing	30°46'42"	107°34'46"	400	CQ	II	6x
13	H040	Fuling, Chongqing	29°44'03"	107°22'11"	190	CQ	II	6x
14	H056	Jiangjing, Chongqing	28°49'43"	106°19'50"	350	CQ	II	6x
15	H057	Dazu, Chongqing	29°40'58"	105°30'36"	400	CQ	II	6x
16	Chonggao	Chongqing	-	-	-	CQ	II	6x
17	H001	Leshan, Sichuan	29°14'05"	103°15'36"	500	CDP	II	6x
18	H003	Yaan, Sichuan	30°01'12"	103°02'04"	670	CDP	II	6x
19	H004	Yaan, Sichuan	30°10'46"	103°13'12"	750	CDP	II	6x
20	H006	Hongya, Sichuan	29°53'20"	103°22'31"	540	CDP	II	6x
21	H009	Hongya, Sichuan	29°53'51"	103°22'25"	480	CDP	II	6x
22	H013	Qionglai, Sichuan	30°23'10"	103°27'36"	520	CDP	II	6x
23	H014	Dayi, Sichuan	30°33'56"	103°30'20"	540	CDP	II	4x
24	H016	Meishan, Sichuan	36°25'00"	103°51'12"	465	CDP	I	4x
25	H017	Leshan, Sichuan	29°32'15"	103°49'05"	390	CDP	I	6x
26	H019	Yaan, Sichuan	29°57'26"	103°06'38"	540	CDP	II	6x
27	H054	Leshan, Sichuan	29°06'28"	104°00'27"	340	CDP	II	6x
28	Guangyi	Guangxi	-	-	-	CDP	III	6x
29	Yaan	Yaan, Sichuan	29°59'48"	103°01'33"	620	CDP	III	6x
30	H026	Ningnan, Sichuan	27°30'50"	102°45'25"	1200	YL	II	6x
31	H028	Ningnan, Sichuan	27°08'28"	102°38'36"	1350	YL	II	4x
32	H029	Ningnan, Sichuan	26°54'12"	102°53'56"	710	YL	III	4x
33	H048	Qiaojia, Yunnan	26°53'51"	102°56'46"	920	YL	I	6x
34	H049	Qiaojia, Yunnan	26°56'18"	102°53'45"	680	YL	I	6x
35	H050	Ningnan, Sichuan	26°53'12"	102°54'58"	670	YL	III	6x
36	H051	Ningnan, Sichuan	26°53'32"	102°54'26"	710	YL	II	6x
37	H058	Panzhihua, Sichuan	26°45'07"	101°50'31"	1200	YL	II	6x

**†** Morphological types are divided according to Chen Y.X. [2]. Ploidy+ are divided according to He L.F. [34]. GZ: Guizhou; CQ: Chongqing; CDP: Chengdu Plain; YL: Yunnan and Liangshan.

### 3.2. DNA Extraction

DNA was extracted from fresh young leaves by the cetyltrimethylammonium bromide (CTAB) protocol [35]. The quality and concentration of the extracted DNA were determined by comparing the sample with known standards of lambda DNA on 1% (w/v) agarose gels quantified using a Bio-Rad gel imaging system. The isolated genomic DNA was diluted to 10 ng/μL and stored at −20 °C until use. 

### 3.3. Primer Selection and PCR Amplification

All SCoT primers were synthesized by Shanghai Sangon Biological Engineering Technology and Services (Shanghai, China). Seventy-five primer sets were initially screened for polymorphism and reproducibility using three varieties of whipgrass. Each 20 μL amplification reaction consisted of 3.0 μL of template DNA (10 ng/μL), 0.8 μM primer (10 μM), 0.2 μL of Taq polymerase (2.5 U/μL), 6 μL of sterile distilled water and 10 μL of 2× Taq PCR MasterMix (Tiangen Biotech, Beijing, China). PCR reactions were performed using a Bio-Rad icycler and the following procedure: an initial denaturation step at 94 °C for 5 min, followed by 35 cycles of 94 °C for 1 min, 51 °C for 1 min, and 72 °C for 2 min and a final extension at 72 °C for 5 min. The PCR amplifications for all primers were processed using the same procedure. All amplified products were resolved by electrophoresis on 1.5% agarose gels containing 0.5 μg/mL ethidium bromide in 0.5× TBE buffer. Images of the banding patterns were acquired under UV light using a Bio-Rad gel imaging system.

### 3.4. Data Scoring and Statistical Analysis

No assumptions about the genetic nature of the SCoT DNA fragments/bands (designated as alleles from this point forward) were made due to the aneu-polyploid nature of whipgrass and the absence of segregation analysis [36]. Therefore, unequivocally scorable bands were scored manually as either present (1) and absent (0). Each band was treated as an independent character regardless of its intensity and used to create a matrix to estimate the variables listed below.

The discriminatory power of the SCoT primer sets was evaluated based on the following eight parameters: the total number of bands (TNB), the number of polymorphic bands (NPB), the percentage of polymorphic bands (PPB), the polymorphism information content (PIC), the genotype index (GI), the resolving power (Rp), the marker index (MI) and Shannon’s diversity index (H). The polymorphism information content (PIC) for each SCoT marker was calculated with the formula described by Roldan-Ruiz* et al.* [21]: PIC*_i_* = 2*f_i_*(1 − *f_i_*) where PIC*_i_* is the polymorphic information content of marker *i*, *f_i_* the frequency of the marker bands which were present, and (1 − *f_i_*) the frequency of marker bands which were absent. PIC values for dominant marker bands such as SCoT markers have a maximum of 0.5 for* f_i_* = 0.5 [22]. PIC values were used to calculate a primer index, which was generated by adding the PIC values of all markers amplified by the same primer. GI reveals the proportion of genotype profiles to the total tested materials studied per assay [37]. The band informativeness (Ib) was estimated using the following equation: Ib = 1 − (2 × |0.5 − p_i_|), where p_i_ is the proportion of the varieties or genotypes containing the band [38]. Rp was measured using the following equation: Rp = ΣIb. MI was determined using the following equation: EMR × DI, where the EMR (Effective Multiplex Ratio) was the number of polymorphic markers generated per assay and the DI (Diversity Index) was the average PIC value [39]. Shannon’s diversity index (H) was calculated using the following equation: H = −∑*f_i_*Ln*f_i_*, where “*f_i_*” is the frequency of an amplified band across all samples. Pearson correlation coefficients were calculated between the last five parameters (PIC, Rp, MI, GI and H). 

Nei and Li’s GS [40] and the unweighted pair group method with arithmetic mean (UPGMA) were used to perform clustering analysis. The clustering analysis was tested by bootstrapping analysis to assess the robustness of the dendrogram topology using NTSYS-pc 2.10 software [41]. Principal coordinate analysis (PCoA) was also performed to determine the location relationship of the 37 accessions in three dimensions. Furthermore, a Mantel test with 10,000 permutations was conducted using the same software to determine the extent of correlation, if any, between the genetic distance (GD = 1 − GS) and the geographical distance (in kilometers) [42].

The Bayesian-based clustering method was applied to delineate the clusters of genetically similar accessions using STRUCTURE software version 2.3.3 [18,43]. An admixture model and an allele frequencies-correlated model were adopted without prior assumptions concerning the population. The value of K was set from 1 to 8. Ten independent runs were performed, each with a Markov Chain Monte Carlo (MCMC) of 100,000 repetitions following a burn-in period of 50,000 iterations [17]. Default values were used for all other parameters. For the chosen K value, the run that had the highest likelihood estimate was adopted to assign individuals to clusters. To identify the optimal value of K, the STRUCTURE output file was implemented in Structure Harvester [44]. The 10 runs with the highest LnP(D) and/or ΔLnP(D) values for the selected K-value were retained, and their admixture estimates were averaged using CLUMPP 1.1 [45]. The run with the maximum likelihood was applied to subdivide the tested accessions into different subgroups using a membership probability threshold of 0.60 [46]. Accessions with less than 0.60 membership probabilities were retained in the admixed group (AD). The results were visualized using DISTRUCT 1.1 [47].

The germplasm collections studied here were artificially grouped into four geographic groups. The geographical distances between accessions within the groups varied greatly from tens to hundreds of kilometers. It is difficult to analyze the variance components and their significance levels of genetic variation within and between geographic groups using common software under the Hardy-Weinberg equilibrium assumption. To avoid the complications mentioned above, genetic divergence was analyzed using Shannon’s diversity index, analysis of molecular variance (AMOVA) and Bayesian inference. The magnitude of the genetic variation was determined for each geographical group using Shannon’s diversity index [48]. Shannon’s diversity index is frequently applied in dominant marker data analysis because the index is insensitive to the potential introduction of bias due to undetectable heterozygosity. Total diversity was calculated using the Shannon index with the following equation: H_W_ = −∑*f_i_*Ln*f_i_*, where “*f_i_*” is the frequency of an amplified band across all samples. The Shannon index within a subset of data (a geographic group) can be calculated using the following equation: H_zone_ = −∑*f_i_*Ln*f_i_*, where “*f_i_*” is the frequency of an amplified band within a subset. The average diversity between different groups was calculated using the following equation: H_A_ = H_zone_ = ∑H_zone_/n, where *n* is the number of groups. Thus, H_A_ is the average group diversity over *n* groups. The intra- and inter-group diversity components were calculated as H_A_/H_W_ and (H_W_ − H_A_)/H_W_, respectively. To compare the levels of diversity detected by different primer sets, the total Shannon diversity was calculated separately for each primer set.

AMOVA based on a Euclidean squared distance matrix was hierarchically calculated to estimate the allocation of genetic variation among and within regional groups using GenAlEx version 6.5 [32]. AMOVA components of variance include Φ_PT_, an analogue of F_ST_ [49]. The significance of the different components of variance was tested with 9999 random permutations. 

We compared the Shannon index and AMOVA results for the population genetic structure with allele-frequency estimates calculated using HICKORY software 1.1 [19]. HICKORY employs a Bayesian approach to estimate *θ^B^* using dominant markers; HICKORY does not assume Hardy-Weinberg equilibrium. Its *f* and *θ^B^* are equivalent to the inbreeding coefficient (F_IS_) and the fixation index (F_ST_) of F-statistics, respectively. The Bayesian estimator of genetic diversity was calculated for each of the following four models: (1) the *full* model (with non-informative priors for *f* and *θ^B^*); (2) *f* = 0 (assumes no inbreeding); (3) *θ^B^* = 0 (assumes no population structure); and (4) *f*-free (allows for the incorporation of uncertainty concerning *f* into the analysis). We conducted several runs with default sampling parameters (burn-in = 50,000, sample = 250,000, thin = 50) to ensure model convergence. The deviance information criterion (DIC) was used to estimate the fit between the data and a particular model and to choose among models [50].

## 4. Conclusions

In summary, the results of our study demonstrate a relatively high level of genetic diversity and low levels of genetic differentiation among geographical groups in whipgrass germplasm. The selected primer sets, which revealed high polymorphism among the accessions, might be applied to other closely related species such as* H. altissima* and *H. sibirica*. Incorrect identification and the misplacement of labels remain serious problems in maintaining germplasm. This study demonstrates that SCoT analysis is a simple, effective and reliable technique for identifying and maintaining warm-season grass germplasm and could have applications in the assessment of genetic variability in collections of unknown origin, breeding stocks, and/or plant variety protection.
